# Publisher Correction: Co-opted transposons help perpetuate conserved higher-order chromosomal structures

**DOI:** 10.1186/s13059-020-1944-4

**Published:** 2020-02-07

**Authors:** Mayank N. K. Choudhary, Ryan Z. Friedman, Julia T. Wang, Hyo Sik Jang, Xiaoyu Zhuo, Ting Wang

**Affiliations:** The Edison Family Center for Genome Sciences & Systems Biology, Department of Genetics, Washington University, 4515 McKinley Avenue, Campus Box 8510, St. Louis, MO 63110 USA

**Publisher Correction to: Genome Biol**

**https://doi.org/10.1186/s13059-019-1916-8**

Following publication of the original paper [1], an error was reported in the processing of Fig. 2. The correct Fig. 2 is supplied below and the original article [1] has been corrected. The publishers apologize for the error.

**Fig. 2:**
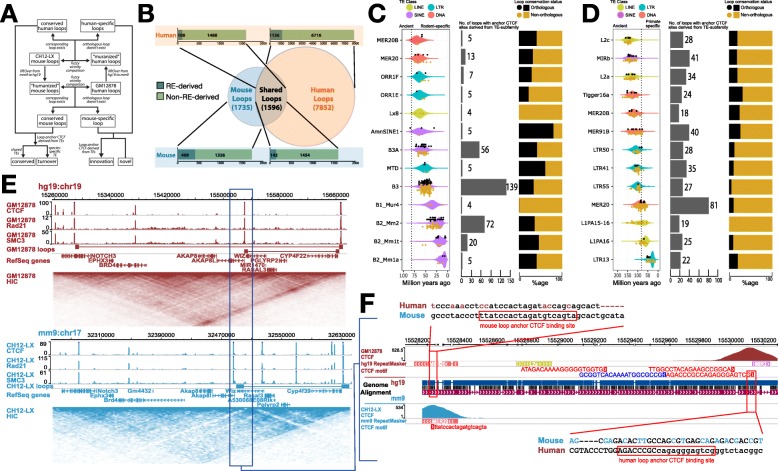
Contribution of TEs to the conservation landscape of human and mouse loops. **a** Flowchart describing the methodology used to annotate loop orthology. **b** Venn diagram representing the various classes of chromatin loops based on their orthology and bar plots showing the contribution of REs to anchor CTCFs of each class of loops. **c** Age distribution and age of individual TEs that contribute loop anchor CTCF sites (black dots for orthologous loops; gold dots for non-orthologous loops) (left), total contribution to loop anchor CTCF sites (middle), distribution of orthologous and non-orthologous loops (right) derived from the top 13 TE subfamilies in mouse and **d** humans. Estimated primate/rodent divergence time (82 million years ago) is from Meredith et al. [47]. **e** Contact maps representing a conserved chromatin loop in a syntenic region between human and mouse. **f** A MER20 transposon insertion provides a redundant CTCF motif that helps in maintaining the conserved 3D structure in mouse via CTCF binding site turnover with remnants of the ancestral CTCF motif, well conserved in most non-rodent mammals (Additional file 1: Figure S2), still seen in the mouse genome
