# Supplementary material for: Co-opted transposons help perpetuate conserved higher-order chromosomal structures
Source: Genome Biol. 2020 Jan 24;21:16. doi: 10.1186/s13059-019-1916-8 (PMC6979391; doi:10.1186/s13059-019-1916-8)
Supplement: Supplementary file 5 — Review History. This file contains reviewers comments and author responses. [file 13059_2019_1916_MOESM5_ESM.docx]

Review History

**First round of review**

**Reviewer 1**

**Are you able to assess all statistics in the manuscript, including the appropriateness of statistical tests used?**

Yes, and I have assessed the statistics in my report.

**Comments to author:**

Transposable elements (TEs) driven dispersal of regulatory elements has long been considered as a fundamental and widespread mechanism of genome evolution. In this manuscript, the authors explored this role of TEs in the context of 3D genome organization by analyzing the contribution of TEs in the anchors of species-specific and conserved loops. In addition to the involvement of TEs in the species-specific structures, the authors found that TEs partially contribute to the conserved loops by providing redundant anchor motifs, which revealed a role of TEs in maintaining the ancestral genome structures. In all, this study is straightforward and provides a new function on the roles of TEs in genome evolution and 3D genome architecture. The TE-derived anchor points, species-specific and shared loops provide a valuable resource to the community. I have however a few concerns:

1.     The authors provided two examples to support their hypothesis that TE may provide redundant CTCF sites in addition to the ancestral CTCF motifs. These examples are convincing, although limited in numbers. Can the authors provide statistics on whether there are degraded ancestral CTCF motifs enriched around TE-derived conserved anchors, to see if this is a general phenomenon?
2.     This study only analyzed one cell line from mouse. It would be helpful to add at least one more mouse cell line to ensure the conclusions are independent of chosen cell lines.
3.     Some statements of the evolutionary impact of TE should be used with caution and should not be over-stated. For example, "Upon deletion of this TE candidate, the local sequence configuration most likely resembled that of the pre-TE insertion, ancestral genome". This is possible but is hard to be proven and therefore should be acknowledged as a speculation. Same is true for "To our knowledge, this is the first-ever example where the evolutionary impact of a TE-derived CTCF site is experimentally demonstrated".
4.     It is very interesting that in mouse, B2 dominates TE-derived CTCF sites, while in humans more classes of TEs are involved. Can the authors speculate why repetitive elements are enriched for CTCF sites, and are there any biological reasons why some elements harbor CTCF sites and while others do not? I could imagine TE elements harbor TF binding sites, but it is a bit difficult to imagine that retroviruses need CTCF.  

Minor points:
5.     Figure 3D, consider using differential Hi-C map to make the difference clearer.
6.     Supplementary Figure 2, 4 and 5: the figures are complex and some texts are too small to read. Consider simplifying the figure.
7.     Some conclusions in the paper are repeated multiple times.

**Reviewer 2**

**Are you able to assess all statistics in the manuscript, including the appropriateness of statistical tests used?**

No, I do not feel adequately qualified to assess the statistics.

**Comments to author:**

Transposon elements (TEs) are known to play an important role in tinkering with 1D and in shaping 2D and 3D genomes. In this paper, the authors investigate the role of transposons in conserving higher-order 3D chromosomal structures. First, the authors classify orthologous and non-orthologous (species-specific) loops for humans and mice based on published Hi-C or ChIA-PET data. The orthologous loops derived by transposable elements (TEs) in various subfamilies and ages are extensively analyzed and compared between humans and mice. Second, the authors use CRIPSR-cas9 to delete two TE-derived CTCF sites which either replace the function of a lost ancestral CTCF site, or reinforce conserved genome architecture as a redundant loop anchor (turnover events). Finally, the authors show that TEs involved in loop anchor turnover have lower methylation levels than the non-involved ones by comparing the methylation-associated C-to-T and G-to-A mutation rates. In summary, the authors used public and in-house-generated data to probe the role of TEs in 3D genome dynamics and relates TEs with CTCF binding sites (thereby higher-order chromosomal structures). While majority of the findings do not appear to be novel, it is worthy publishing the punch line that TE-mediated CTCF site turnover appears to be important for conserving 3D genome architecture.

Major points:
(1). Given the known role of orientation of CTCF sites and directional CTCF binding in shaping the 3D genome (Rao 2014; Guo 2015), it would be much stronger to frame the current finding of CTCF binding site turnover by TEs in term of its orientation.
(2). It appears the opposite direction of the CTCF motif in the reverted mouse syntenic region is not actually inverted between mouse and human. It is conserved!? The presentations in Figure 2DE and Figure S2BC are confusing. There should be much better ways to straighten it out.
(3). I would like see the Sanger sequencing confirmation and traces for deletion clones of CTCF binding sites even though similar experiments and effects have assayed before.
(4). What will happen if CTCF binding site turnover happened but in an opposite orientation? Do TEs know how to transpose in the same orientation as "degraded" or "decomposed" ones? These needs to be better described.

Minor points:
(1) In line 405, the meaning of "unidentified loop anchor CTCF sites" is not defined well. In fact, only RE-derived loop anchor CTCF site is defined in line 400. The statements from line 402 to line 405 exclude the possibility that "unidentified" means "non-RE derived".
(2) The calculation of false discovery rates in line 435 is not described clearly. In my opinion, simulated sets in mouse should be compared with human features. So what is "the orthology observed between the real CH12 (mouse) and GM12878 (human) features"?
(3) In line 444, the process to pick exactly one orthologous human loop for a corresponding mouse loop is not clearly stated.
(4) In line 462, "after identifying the list of TE-derived CTCF turnover events in humans ...". However, the following lines just describe how to do this.
(5) The authors use Welch's t-Test in line 560, while Student's t-Test in line 562. Note that both tests assume normally distributed data. However, the random variables here are discrete (intradomain or interdomain). I suggest to use Fisher's exact test instead.
(6) There is an error in line 102. 1136 out of 6716? Not out of 6716.

**Authors Response**

**Point-by-point responses to the reviewers’ comments:**

=== Reviewer #1: ===

Transposable elements (TEs) driven dispersal of regulatory elements has long been considered as a fundamental and widespread mechanism of genome evolution. In this manuscript, the authors explored this role of TEs in the context of 3D genome organization by analyzing the contribution of TEs in the anchors of species-specific and conserved loops. In addition to the involvement of TEs in the species-specific structures, the authors found that TEs partially contribute to the conserved loops by providing redundant anchor motifs, which revealed a role of TEs in maintaining the ancestral genome structures. In all, this study is straightforward and provides a new function on the roles of TEs in genome evolution and 3D genome architecture. The TE-derived anchor points, species-specific and shared loops provide a valuable resource to the community. I have however a few concerns:

1. The authors provided two examples to support their hypothesis that TE may provide redundant CTCF sites in addition to the ancestral CTCF motifs. These examples are convincing, although limited in numbers. Can the authors provide statistics on whether there are degraded ancestral CTCF motifs enriched around TE-derived conserved anchors, to see if this is a general phenomenon?

*(1.1) We thank the reviewer for the request. We find that out of 127 TE turnover events in mouse, 32 human loop anchor CTCF motifs (regions) are deleted in mouse, 17 human loop anchor CTCF motifs are degraded (beyond CTCF binding) in mouse, 65 human loop anchor CTCF motifs are still present in mouse but either have lowered binding or TE-derived mouse loop anchor CTCF motif is either (i) downstream of the constitutive left loop anchor, or (ii) upstream of the constitutive right loop anchor, leading to it being encountered by the extrusion complex before the putative ancestral loop anchor CTCF motif.

In humans, we find that out of 93 turnover events, 30 mouse loop anchor CTCF motifs (regions) are deleted in humans, 16 human loop anchor CTCF motifs are degraded (beyond CTCF binding) in mouse, 16 human loop anchor CTCF motifs are still present in mouse but either have lowered binding or TE-derived mouse loop anchor CTCF motif is either (i) downstream of the constitutive left loop anchor, or (ii) upstream of the constitutive right loop anchor, leading to it being encountered by the extrusion complex before the putative ancestral loop anchor CTCF motif.

In conclusion, we were able to identify the fate of 90% (114 out of 127) and 67% (62 out of 93) of the putative ancestral loop anchor CTCF motifs in mouse and humans respectively.*

2. This study only analyzed one cell line from mouse. It would be helpful to add at least one more mouse cell line to ensure the conclusions are independent of chosen cell lines.

*(1.2) One of the biggest impediments to performing this analysis is the unavailability of high resolution in situ Hi-C maps. Moreover, we require CTCF, Rad21 and/or SMC3 ChIP-seq datasets for the respective cell lines to accurately annotate loop anchors, making it even more challenging. For the purposes of this study we utilized datasets from Rao et al (2014) which profiled some of the most commonly used cell lines.

To address the reviewer’s request, we now include 2 more mouse cell lines—ESCs (embryonic stem cells) cultured in FBS and NSCs (neural stem cells) from Gain of CTCF-Anchored Chromatin Loops Marks the Exit from Naive Pluripotency by Aleksandra Pękowska et al. (2018). Loop calling was performed by Pekowska et al. using 2 biological replicates comprising of 1.23B (ESCs) and 1.28B (NSCs) total reads. Pekowska et al. annotated 3868 and 8444 loops calls in ESCs and NSCs respectively. Using these loop calls, we adapted JuicerTools’ MotifFinder, to identify loop anchor CTCF motifs (as described in Rao et al 2014). We found 3056 and 6116 unique loop anchors with a discernible CTCF motif by following the procedure as described in our updated Methods. Out of these, 691 (22.6%) and 773 (12.6%) motifs were derived from repetitive elements (equivalent to Fig 1B). These RE-derived loop anchor CTCF motifs tether 722 out of 2698 analyzed loops (26.8%) in ESCs and 976 out of 6393 analyzed loops (15.3%) in NSCs (equivalent to Fig 1A). We also see that the relative contribution of the various classes of REs is fairly consistent to what was observed in CH12-LX cell lines. This analysis shows the robustness of the contribution of repeats (and TEs) to loop anchor CTCF sites in various cell lines as well as data generated and analyzed by different labs using different methodologies. We have now incorporated these results as part of an updated Fig 1:*

3. Some statements of the evolutionary impact of TE should be used with caution and should not be over-stated. For example, "Upon deletion of this TE candidate, the local sequence configuration most likely resembled that of the pre-TE insertion, ancestral genome". This is possible but is hard to be proven and therefore should be acknowledged as a speculation. Same is true for "To our knowledge, this is the first-ever example where the evolutionary impact of a TE-derived CTCF site is experimentally demonstrated".

*(1.3) We apologize and have now edited the manuscript to reflect the change:

1. Upon deletion of this TE candidate, the local sequence configuration probably resembled that of the pre TE-insertion, ancestral genome
2. Thus, experimentally demonstrating the evolutionary impact of a TE-derived CTCF site.*

4. It is very interesting that in mouse, B2 dominates TE-derived CTCF sites, while in humans more classes of TEs are involved. Can the authors speculate why repetitive elements are enriched for CTCF sites, and are there any biological reasons why some elements harbor CTCF sites and while others do not? I could imagine TE elements harbor TF binding sites, but it is a bit difficult to imagine that retroviruses need CTCF.

*(1.4) We thank the reviewer for the excellent question and now discuss this briefly in the main text as well.

The role of CTCF motifs in viral genome regulatory control has been a topic of tremendous interest and vast investigation. In EBV, this control involves direct binding of CTCF across the viral genome and the formation of three-dimensional loops between virus promoters and enhancers (Tempera, I. et al. EBV latency types adopt alternative chromatin conformations. PLoS Pathog 7:e1002180 (2011)). CTCF has also been shown to be important in the regulation of gene expression of a number of human DNA viruses. (Pentland, I. et al. Targeting CTCF to Control Virus Gene Expression: A Common Theme amongst Diverse DNA Viruses. Viruses 7:3574-85. (2015)). CTCF also plays a critical role in epigenetic regulation of viral gene expression to establish and/or maintain a form of latent infection that can reactivate efficiently. (Lee, JS. et al. CCCTC-binding factor acts as a heterochromatin barrier on herpes simplex viral latent chromatin and contributes to poised latent infection. mBio 9:e02372-17. (2018)).

Among various functions of CTCF is its role in transcriptional activation. Moreover, one can speculate that having a CTCF motif can not only help in maintaining viral genome confirmation but can also help insulate the chromatin activity of the neighborhood wherein the virus inserts into the host genome. It may also increase the chances of long-range interactions taking place which can sometimes bring in other TFs and/or polymerase, leading to enhanced transcription at the site of viral integration. Recently evidence has also shown that HTLV-1, forms loops between the provirus and host genome, and alters expression of proviral and host genes. (Satou, Y. et al. The retrovirus HTLV-1 inserts an ectopic CTCF-binding site into the human genome. Proc Natl Acad Sci USA. 113(11):3054-3059 (2016)). CTCF has also been shown to promote HSV-1 lytic transcription by facilitating the elongation of RNA Pol II and preventing silenced chromatin on the viral genome (Lang, F. et al. CTCF interacts with the lytic HSV-1 genome to promote viral transcription. Sci. Rep. 7, 39861 (2017)).

On the other hand, B2 SINEs are derived from tRNA genes. Recent evidence has shown that human tRNA genes can function as chromatin insulators, both in CTCF-dependent and CTCF-independent manners. (Raab, JR. et al. Human tRNA genes function as chromatin insulators. EMBO J. 31:330-350 (2012)). Using silencer blocking, transgene protection and repressor blocking assays, Raab et al., experimentally demonstrate that some tRNA gene containing fragments can function as barrier insulators in human cells. Mouse tRNA genes have also been shown to possess classical insulator activity and the potential to function as boundary elements (Ebersole, T. et al. tRNA genes protect a reporter gene from epigenetic silencing in mouse cells. Cell Cycle 10, 2779–2791 (2011)). Moreover, CTCF-binding enrichment in B2 SINEs in mouse has been studied before and repeat-driven dispersal of CTCF-binding has been shown to be a fundamental, ancient, and still highly active mechanism of genome evolution in mammalian lineages. (Schmidt, D. et al. Waves of retrotransposon expansion remodel genome organization and CTCF binding in multiple mammalian lineages. Cell 148, 335–348 (2012)).*

Minor points:
5. Figure 3D, consider using differential Hi-C map to make the difference clearer.

*(1.5) We thank the reviewer for the suggestion. We provide a differential Hi-C map plotted by calculating the difference between the VC_sqrt normalized observed interaction scores of the KO and the WT contact maps. We plot differential normalized interaction scores for which fold change (KO/WT) > 1.45 or (WT/KO) > 1.45 (fold cut-off), and normalized interaction score of >0.75 (score cut-off) in KO and WT. Blue and green arrows represent the loop’s original right anchor (mainly used in WT) and the turned-over loop anchor (mainly used in KO), respectively. Focal blue enrichment suggests loss of loop in the KO as compared to the WT. Similarly, focal red enrichment suggests gain of loop in KO as compared to the WT.

To increase the resolution of our Hi-C2 contact map for Fig 3D, we sequenced our Hi-C2 libraries deeper and provide an enhanced Fig 3D. Please find below a comparison of the old (left) and new (right) Fig 3D:* *(old) ——> (new)* *The features are especially striking when more colors are added to the higher-resolution Hi-C2contact map visualization as shown below:*

*We now provide the differential HiC map as Supplementary Figure 7B, and the HiC2 contact map in different color as Supplementary Figure 7A.*

6. Supplementary Figure 2, 4 and 5: the figures are complex and some texts are too small to read. Consider simplifying the figure.

*(1.6) We have increased the size of the labels and simplified the text as recommended by the reviewer.*

7. Some conclusions in the paper are repeated multiple times.

*(1.7) We apologize and have revised the text as per the reviewer’s suggestion.*

=== Reviewer #2: ===

Transposon elements (TEs) are known to play an important role in tinkering with 1D and in shaping 2D and 3D genomes. In this paper, the authors investigate the role of transposons in conserving higher-order 3D chromosomal structures. First, the authors classify orthologous and non-orthologous (species-specific) loops for humans and mice based on published Hi-C or ChIA-PET data. The orthologous loops derived by transposable elements (TEs) in various subfamilies and ages are extensively analyzed and compared between humans and mice. Second, the authors use CRIPSR-cas9 to delete two TE-derived CTCF sites which either replace the function of a lost ancestral CTCF site, or reinforce conserved genome architecture as a redundant loop anchor (turnover events). Finally, the authors show that TEs involved in loop anchor turnover have lower methylation levels than the non-involved ones by comparing the methylation-associated C-to-T and G-to-A mutation rates. In summary, the authors used public and in-house-generated data to probe the role of TEs in 3D genome dynamics and relates TEs with CTCF binding sites (thereby higher-order chromosomal structures). While majority of the findings do not appear to be novel, it is worthy publishing the punch line that TE-mediated CTCF site turnover appears to be important for conserving 3D genome architecture.

Major points:
(1). Given the known role of orientation of CTCF sites and directional CTCF binding in shaping the 3D genome (Rao 2014; Guo 2015), it would be much stronger to frame the current finding of CTCF binding site turnover by TEs in term of its orientation.

*(2.1) We thank the reviewer for this important suggestion.

In mouse, 127 turnover events affecting 123 loops (4 loops had both loop anchors turned-over) were mediated by 124 unique loop anchors (3 turned-over loop anchors tethered 2 loops each). Out of the 124 unique loop anchors, 61 events represent turnover of the left loop anchor and 63 events represent turnover of the right loop. In terms of the orientation of the CTCF motif—for the 61 left loop anchor turnover events, 53 were +ve and 8 were -ve, and for the 63 right loop anchor turnover events, 45 were -ve and 18 were +ve (Chi-square test, p-value=5.3x10-11).*

| ***mm9 CH12-LX (n=124)*** | *Left Loop Anchor* | *Right Loop Anchor* |
| --- | --- | --- |
| *+ve CTCF motif* | *53* | *18* |
| *-ve CTCF motif* | *8* | *45* |

*In humans, 93 turnover events affecting 89 loops (4 loops had both loop anchors turned-over) were mediated by 84 unique loop anchors (1 turned-over loop anchor tethered 3 loops, and 7 loop anchors tethered 2 loops each). Out of the 84 unique loop anchors, 43 events represent turnover of the left loop anchor and 41 events represent turnover of the right loop. In terms of the orientation of the CTCF motif—for the 43 left loop anchor turnover events, 43 were +ve and 0 were -ve, and for the 41 right loop anchor turnover events, 40 were -ve and 1 was +ve (Chi-square test, p-value=3.6x10-19).*

| ***hg19 GM12878 (n=84)*** | *Left Loop Anchor* | *Right Loop Anchor* |
| --- | --- | --- |
| *+ve CTCF motif* | *43* | *1* |
| *-ve CTCF motif* | *0* | *40* |

*These results further lend credence to the loop extrusion model and suggest that TE exaptation is more likely when the orientation of the inserted TE (and the underlying CTCF motif provided) is compatible with the local loop structure. We now include these results in the main text.*

(2). It appears the opposite direction of the CTCF motif in the reverted mouse syntenic region is not actually inverted between mouse and human. It is conserved!? The presentations in Figure 2DE and Figure S2BC are confusing. There should be much better ways to straighten it out.

*(2.2) We thank the reviewer for the keen observation. Indeed, the turned-over CTCF motif is in the opposite orientation of the putative ancestral CTCF motif. While most loops from HiC data are anchored at convergent CTCF motifs, a small fraction of loops are derived from divergent and tandem left/right CTCF motifs (explained by coiled-loop structure as described in Tang et al., 2015) as well. We have provided more details regarding this in Response (2.1) and (2.4).

To remove ambiguity, we have replaced Fig 2D with a different example of a CTCF turnover site in mouse and made changes in the text to reflect the same.*

(3). I would like see the Sanger sequencing confirmation and traces for deletion clones of CTCF binding sites even though similar experiments and effects have assayed before.

*(2.3) We thank the reviewer for this request. The precise genotyping was included as a new Supplementary Figure 5, and included below for the reviewer’s reference:*

 *We have also submitted the trace (.ab1) files (CRISPR_Genotype_SangerTrace.zip) along with our revision.*

(4). What will happen if CTCF binding site turnover happened but in an opposite orientation? Do TEs know how to transpose in the same orientation as "degraded" or "decomposed" ones? These needs to be better described.

*(2.4) CTCF is a multi-functional protein that can function as a transcriptional activator, a repressor or an insulator protein. Moreover, it plays a role in tethering loops and can also recruit other transcription factors while being bound to chromatin domain boundaries. Hence, depending on its underlying function, its orientation may or may not matter. Additionally, we also know that a subset of loops are formed with either divergent CTCF motifs as well as tandem left or tandem right pairs of CTCF motifs (Rao, S. S. et al. A 3D map of the human genome at kilobase resolution reveals principles of chromatin looping. Cell 159, 1665–1680 (2014), Tang, Z. et al. CTCF-mediated human 3D genome architecture reveals chromatin topology for transcription. Cell 163, 1611–1627 (2015)). Hence, a CTCF binding site turnover event resulting in an opposite orientation motif, may or may not interfere with its underlying function.

Regarding the latter, TEs do not necessarily ‘know how to transpose in the same orientation as “degraded” or “decomposed” ones’. Assigning purpose to TE transposition is an anthropomorphic tendency. Evolution, and turnover for that matter, does not happen with a purpose in mind. TEs insert pseudorandomly (according to their target-site preferences, which can include open chromatin regions usually found close to loop anchor CTCF sites) in the genome and as long as they are not deleterious and can provide the organism the same fitness (neutral evolution/genetic drift), or if they can provide the organism a fitness advantage, they are selected for and perpetuated. In the former case, during the course of millions of years of evolution the original ancestral CTCF site can lose selection pressure and degrade away as the TE-derived CTCF motif becomes the putative CTCF binding site and starts being selected for.*

Minor points:
(1) In line 405, the meaning of "unidentified loop anchor CTCF sites" is not defined well. In fact, only RE-derived loop anchor CTCF site is defined in line 400. The statements from line 402 to line 405 exclude the possibility that "unidentified" means "non-RE derived".
*(2.5.1) We apologize for the confusion. “Unidentified” here does not mean non-RE derived. “Unidentified loop anchor CTCF sites” are loop anchors wherein HiCCUPS motif finder tool was unable to identify a putative loop anchor CTCF motif. To clarify the types of loops that were used for the analysis, we’ve segmented all the loops as shown below:
Cat. Left Loop Anchor CTCF site Right Loop Anchor CTCF site Used for Analysis*

| ***Cat.*** | ***Left Loop Anchor CTCF site*** | ***Right Loop Anchor CTCF site*** | ***Used for Analysis*** |
| --- | --- | --- | --- |
| *1* | *Identified* | *Identified* | *Yes* |
| *2* | *Identified and non-RE-derived* | *Unidentified* | *No* |
| *3* | *Identified and RE-derived* | *Unidentified* | *Yes* |
| *4* | *Unidentified* | *Identified and non-RE-derived* | *No* |
| *5* | *Unidentified* | *Identified and RE-derived* | *Yes* |
| *6* | *Unidentified* | *Unidentified* | *No* |

*The RE-derivation status of unidentified loop anchor CTCF sites of loops belonging to category 2,4 and 6 is ambiguous and hence not included in the overall analysis. Our intention was to perform the analysis on loops whose loop anchor CTCF sites we can unambiguously and definitively say were either derived from REs or not.*

(2) The calculation of false discovery rates in line 435 is not described clearly. In my opinion, simulated sets in mouse should be compared with human features. So what is "the orthology observed between the real CH12 (mouse) and GM12878 (human) features"?

*(2.5.2) We apologize for the potential confusion. The reviewer is absolutely correct in stating that “simulated sets in mouse should be compared with human features”. In fact, that is precisely what we did. We simulated sets in mouse, humanized those simulated mouse features (by using liftover) and then compared their orthology with human features. We have improved clarity in the updated Methods.*

(3) In line 444, the process to pick exactly one orthologous human loop for a corresponding mouse loop is not clearly stated.

*(2.5.3) Too add more detail,
Finally, we curated the list by considering genic, epigenomic and transcriptomic synteny to pick exactly one orthologous human loop to a corresponding mouse loop, to enlist 1596 high-confidence orthologous peak calls (Table S1.1)
We required that the gene structure (i.e. genes contained within the loops as well as just outside the loops anchors) around the loop was equivalent across humans and mouse to call a loop conserved.*

(4) In line 462, "after identifying the list of TE-derived CTCF turnover events in humans ...". However, the following lines just describe how to do this.

*(2.5.4) The “following lines” describe the procedure behind selecting candidate TEs for validation as presented in the manuscript. We have explained in detail how TE-derived loop anchor CTCF sites were identified (please see Line 397 for section titled “Loop anchor CTCF–RE intersection”), and for mapping human and mouse orthologous loops, please see Line 420 for section titled “Loop orthology check”. After identifying corresponding human-mouse orthologous loops, we determined TE-mediated CTCF turnover events by looking for TE-derived orthologous loops in humans (or mouse) that were discordant for TEs in mouse (or humans).*

(5) The authors use Welch's t-Test in line 560, while Student's t-Test in line 562. Note that both tests assume normally distributed data. However, the random variables here are discrete (intradomain or interdomain). I suggest to use Fisher's exact test instead.

*(2.5.5) We thank the reviewer for the thoughtful suggestions and attention to detail. We performed unequal variances t-Test (Welch’s t-test). We apologize for erroneously mentioning Student’s in the methods text. Moreover, we would like to provide additional explanation regarding our methodology. From the Methods section:

We subsampled the Hi-C2 corresponding to the R1-WT megamap (containing 46M reads) and R1-KO (containing 56M reads) for 5M reads, 10 times to create 10 independent R1-WT and R1-KO mini-maps. For each of these HiC maps, we used the Juicer Tools dump command to extract the raw contact matrix. Intradomain interactions were defined as interaction that (i) originate and terminate in domain 1, or (ii) originate and terminate in domain 2. Interdomain interactions were defined as interactions that originate in domain 1 and terminate in domain 2. We then calculate percentage of cross-domain interactions for each of the mini-maps using the formula: (number of intradomain-interactions)*100 / ((number of intradomain-interactions) + (number of interdomain-interactions)). The percentage of cross-domain interactions were calculated for the target domain as well as a control domain. The distribution of cross-domain interactions across the targeted domain was found to be significantly difference in the KO vs. the WT (t-Test: Two-Sample Assuming Unequal Variances, p-value = 1.40668x10-16). The distribution of cross-domain interactions across a nearby control domain however was not found to be significantly different in the KO vs. the WT (t-Test: Two-Sample Assuming Unequal Variances, p-value = 0.013254165). Raw simulation data and statistics are provided in Table S2.5.

Hence, we only compared the distribution of percentage of interdomain interactions from 10 subsampled Hi-C2 mini-maps across the targeted domain in WT and KO genotype. We found that the distribution of percentage of interdomain interactions was significantly different across the targeted domain in the WT vs the KO. However, across a control domain no such association was found.

Additionally, as per the reviewer’s request, we also performed a Fisher’s Exact test and found that there is a statistically significant association between the type of interaction and genotype when we perform the analysis on the targeted domain (p-value = 0.0185, Fisher’s Exact Test, one-tailed)*

| ***Targeted Domain*** | *Wild-type* | *L1M3f-DKO* |
| --- | --- | --- |
| *Percent interdomain interactions* | *92* | *81* |
| *Percent intradomain interactions* | *8* | *19* |

*Similarly, we find that there isn’t a significant association between the type of interaction and genotype when we perform the analysis on a control domain (p-value = 0.4071, Fisher’s Exact Test, one-tailed).*

| ***Control Domain*** | *Wild-type* | *L1M3f-DKO* |
| --- | --- | --- |
| *Percent interdomain interactions* | *89* | *91* |
| *Percent intradomain interactions* | *11* | *9* |

(6) There is an error in line 102. 1136 out of 6716? Not out of 6716.

*(2.5.6) We thank the reviewer for bringing the error to our attention. It should be 7852 (1136+6716). We have updated the manuscript to reflect the change.*

**Second round of review**

**Reviewer 1**

The authors have addressed my comments and improved the manuscripts with additional datasets and statistic analysis. I only have one minor suggestion:
1.     In revised Fig. 1, the portion of RE-derived loops and anchor CTCF sites in mESCs is significantly higher than other two mouse cell lines and also human cell lines. It would be interesting to point this out and discuss it if possible.

**Reviewer 2**

In the revised manuscript, Choudhary et al. added two new mouse HiC data and analyzed orientation of CTCF-binding-site turnover according to the reviewer’s comments. Although the analyzed data support the loop extrusion model to some extent, it is unclear how TE-mediated CTCF-site turnover appears to be important and what the mechanism for conserving 3D genome architecture. In particular, if TE does not know the orientation of how to integrate into the genome, then the orientation of the carried CTCF-binding-site will not be important. Since the novelty of the paper rests mainly on the conservation of 3D genome architecture by TE, then the whole arguments collapse. In fact, some TE may have specific targeting sequences to integrate into the genome. These TEs may know the orientation to integrate into the genome, and the orientation may not be just anthropomorphic. The evolutionary arguments may be true that one orientation is selected for and the other are selected against. However, these are just arguments and cannot be easily approved. TEs are silenced by piRNA pathways not just by methylation. If silencing machineries have broken parts caused by mutations, TEs become active. Thus, there must be an underlying mechanism for maintaining TE integration orientation which is quintessential for conservation of 3D genome architecture.

According to the new analyzed data of orientation of CTCF sites at the left and right loop anchor in mouse CH12-LX and human GM12878 cell line, the author acknowledge the role of orientation of CTCF sites in shaping the 3D genome, especially “when the orientation of the inserted TE is compatible with the local loop structure”, but if TE is not compatible, what will happened?

The author only deleted two inserted TEs which are convergent or tandem to the adjacent CTCF sites, whether TE will maintain the 3D genome architecture while the inserted TEs are divergent to the adjacent CTCF sites? Moreover, deletion of the L1M3f-derived loop anchor merged two neighboring domains, whether the integration site matters at the TAD boundary were not clearly elucidated.

If ancestral CTCF binding motif is responsible for maintaining the conserved higher order chromosomal structures, whether inserting a younger TE in vivo will model the evolutionary process?

Minor points:

Too or to “Too add more detail”

What’s the “+ve-ve”

Reviewers’ life could be easier if less undecipherable computer jargons and references are required to “known” before understanding well the flow of logic.

The Supplementary Figure 5 does not contain Sanger sequencing traces.

ChIP-seq data should be provided for the deletion of the LTR41-derived CTCF site, as ancestral MER82 has reduced CTCF binding strength compared to the LTR41 in wild-type cells.

**Authors Response**

**Point-by-point responses to the reviewers’ comments:**

=== Reviewer #1: ===

The authors have addressed my comments and improved the manuscripts with additional datasets and statistic analysis. I only have one minor suggestion:

1. In revised Fig. 1, the portion of RE-derived loops and anchor CTCF sites in mESCs is significantly higher than other two mouse cell lines and also human cell lines. It would be interesting to point this out and discuss it if possible.

*(2.1.1) We thank the reviewer for the thoughtful suggestion. We have now highlighted this observation and briefly discussed it in the manuscript:*

*Interestingly, the proportion of RE-derived loops and anchor CTCF sites in mouse ESCs is significantly higher than NSCs and CH12LX cells. This observation could potentially be driven by both: genome-wide demethylation of transposable elements in embryonic stem cells leading to a higher proportion of TE-derived CTCF motifs accessible for CTCF binding as well as fewer chromatin loops observed in mESCs (Pekowska et al., 2018).*

=== Reviewer #2: ===

In the revised manuscript, Choudhary et al. added two new mouse HiC data and analyzed orientation of CTCF-binding-site turnover according to the reviewer’s comments. Although the analyzed data support the loop extrusion model to some extent, it is unclear how TE-mediated CTCF-site turnover appears to be important and what the mechanism for conserving 3D genome architecture. In particular, if TE does not know the orientation of how to integrate into the genome, then the orientation of the carried CTCF-binding-site will not be important. Since the novelty of the paper rests mainly on the conservation of 3D genome architecture by TE, then the whole arguments collapse.

*(2.2.1) We thank the reviewer for the remarks, and apologize for any potentially misleading statement regarding*

*the novelty of our study and the inferred evolutionary events. We want to clarify for the reviewer as well as the readers that there is absolutely no support, neither in theory nor in data, for the idea that TEs know which orientation to insert in so that they can maintain 3D chromatin structure. Neither the mechanism by which TEs contribute to conserved 3D chromatin structure, nor the novelty of our study, is built upon such a presumption.*

*Regarding the novelty of the paper we have carefully described in the Discussion section:*

*Pioneering work in the last decade has extensively outlined this contribution of TEs in shaping gene regulatory networks by depositing TF binding sites in host genomes, leading to the origins of novel phenotypes like innate immunity and pregnancy in mammals. Herein, lies the catch: research to date showcases the role of TEs in bringing novelty and new regulatory functions to the host genome. Hence, TEs have long been considered a source of genetic innovation. However, by comparing topologies instead of raw DNA sequences in this study, for the first time, we have been able to reveal the role of TEs in 3D genome conservation. This seemingly counter­intuitive role of species-specific parasitic sequences in helping maintain ancestral genome architecture is fundamentally different from all current and previous work regarding TEs’ role in gene regulation. This role is mediated by a long-postulated, classic genetic phenomena of binding site turnover—for CTCF in this case. Redundant TE-derived CTCF sites in the vicinity of conserved chromatin anchor/boundary can sometimes take over from the conserved anchor/boundary element, thus slightly shifting the anchor/boundary site while largely maintaining the 3D structure.*

*Hence, TEs contribute to regulatory plasticity by introducing binding site redundancy in their host genomes. In this case, TEs provide redundant CTCF binding sites that contributes to host genome plasticity and helps increase regulatory robustness. We further clarified in the Discussion section:*

*The genome is replete with such events and we have for the first time functionally dissected and validated them in the context of 3D genome conservation, opening the doors up for such investigations in the field for enhancer or promoter turnover events.*

*We feel this is an important conceptual leap and the discovery is fundamentally different from all previous work in the field addressing TEs’ role in gene regulation.*

*Indeed, since the submission of our manuscript, an independent study by Kentepozidou, E., et al. (Clustered CTCF binding is an evolutionary mechanism to maintain topologically associating domains. bioRxiv (2019)) has found that TAD boundaries frequently harbor clusters of CTCF sites that contribute to cohesin stabilization and are critical for the functional stability of higher-order chromatin structure. Moreover, some of the CTCF sites in these clusters are derived from TEs, further appreciating the role of TEs in the maintenance of higher-order chromosomal structures in mammals.*

*Additionally, we would like to clarify, that we do see multiple TE-derived CTCF motifs of either orientations in the vicinity of loop anchors. However, if the orientation of the TE-derived CTCF site is compatible with the local loop structure, it is more likely to anchor and perpetuate the loop.*

In fact, some TE may have specific targeting sequences to integrate into the genome. These TEs may know the orientation to integrate into the genome, and the orientation may not be just anthropomorphic.

*(2.2.2) We apologize for the confusion and would like to take this opportunity to provide a point-by-point clarification. As stated in our previous response section (2.4)*

*Assigning purpose to TE transposition is an anthropomorphic tendency. Evolution, and turnover for that matter, does not happen with a purpose in mind.*

*We meant that TEs do not transpose with a “higher purpose” in mind and assigning said “higher purpose” is a human tendency (anthropomorphic).*

*The reviewer is absolutely correct in stating that “some TE may have specific targeting sequences to integrate into the genome”. Indeed, TEs integrate into host genomes with varying degrees of target site specificity using diverse mechanisms. As mentioned in our previous response section (2.4):*

*TEs insert pseudorandomly (according to their target-site preferences, which can include open chromatin regions usually found close to loop anchor CTCF sites) in the genome and as long as they are not deleterious and can provide the organism the same fitness (neutral evolution/genetic drift), or if they can provide the organism a fitness advantage, they are selected for and perpetuated. In the former case, during the course of millions of years of evolution the original ancestral CTCF site can lose selection pressure and degrade away as the TE-derived CTCF motif becomes the putative CTCF binding site and starts being selected for.TEs do have integration site preferences and that remains an area of active research. In fact, some of the best characterized transposons are the ones commonly used as tools in molecular biology including piggyBac, Sleeping Beauty, etc. At the sequence level, piggyBac prefers to insert into TA-rich regions within a broader GC-rich context (Li, M. A., et al. The piggyBac Transposon Displays Local and Distant Reintegration Preferences and Can Cause Mutations at Noncanonical Integration Sites. Mol Cell Biol. 33(7), 1317–1330 (2013)). Transposons may also display chromatin state-related preferences (Yoshida, J., et al. Chromatin states shape insertion profiles of the piggyBac, Tol2 and Sleeping Beauty transposons and murine leukemia virus, Scientific Reports, (2017)).*

*In the human genome, we observe Alus to be enriched in GC-rich regions and LINE1 elements to be enriched in AT-rich regions (Lander, E., Linton, L., Birren, B. et al. Initial sequencing and analysis of the human genome. Nature 409, 860–921 (2001)). However, it is inaccurate to attribute their observed present-day location to insertion preference as it has been guided by selective forces and genetic drift over millions of years of evolution, shaping the accumulation and distribution of TEs in host genomes (Bourque, G., et al. Ten things you should know about transposable elements. Genome Biology (2018)). More recently, using engineered L1 insertions in 5 human cell lines, it has been shown that L1 insertion positions are indeed located in genomic regions with a high local AT content at a L1 endonuclease consensus cleavage site. (Flasch, D. A., et al. Genome-wide de novo L1 Retrotransposition Connects Endonuclease Activity with Replication. Cell. 177, 837–851 (2019))*

*We respectfully reject the notion that “These TEs may know the orientation to integrate into the genome”. If one were to look at LTR retrotransposons distribution in the human genome, you would see a strong depletion in the sense orientation within human introns. This wouldn’t be because TEs know that they should be inserting in the antisense orientation. In fact, no LTR retrotransposon is known to exhibit insertion preference with regard to the transcribed DNA strand. Instead, sense orientation insertions were selected against due to their propensity to interfere with gene splicing and polyadenylation (Medstrand, P., et al. Retroelement distributions in the human genome: variations associated with age and proximity to genes. Genome Res. 12:1483–95. (2002)).*

*Hence, the landscape of transposons in the host genome is shaped by pre-insertion sequence biases and post-insertion selection. The large majority of TE insertions do not survive in the host genome; those that are present in the host genome are mostly neutral, but a few can be co-opted by the host to serve a host function – this is a process called exaptation. The novel phenomenon we report here, i.e., TE-mediated CTCF site turnover resulting in maintaining conserved 3D chromatin structure, are events distilled through many millions of years of evolution. The novelty is not that TEs purposely insert in a compatible orientation to maintain conserved 3D genome – we categorically reject this hypothesis. The novelty is that the function of these co-opted TEs seems to be to maintain conserved 3D genome, rather than to create species-specific functions (which represents the main theme of all previous discoveries related to TE exaptation).*

The evolutionary arguments may be true that one orientation is selected for and the other are selected against. However, these are just arguments and cannot be easily approved.

*(2.2.3) We agree with the reviewer that evolutionary arguments cannot be easily proved and hence do not make any claims related to this in our original manuscript. Empirically, our data supports that in the event of a CTCF binding site turnover, we see higher number of TE-derived CTCF motifs that maintain the original orientation which supports their selection. We pointed this out in our response section (2.4):*

*TEs insert pseudorandomly (according to their target-site preferences, which can include open chromatin regions usually found close to loop anchor CTCF sites) in the genome and as long as they are not deleterious and can provide the organism the same fitness (neutral evolution/genetic drift), or if they can provide the organism a fitness advantage, they are selected for and perpetuated. In the former case, during the course of millions of years of evolution the original ancestral CTCF site can lose selection pressure and degrade away as the TE-derived CTCF motif becomes the putative CTCF binding site and starts being selected for.*

*Moreover, throughout the genome we did not find significant orientation differences in TE integrations. We are limited by our ability to quantitatively measure the selection pressure, and again we do not claim to do so. However, our data is consistent with a model in which TE insertions do not have orientation bias, whereas co-option of TE-derived CTCF sites for chromatin looping does.*

TEs are silenced by piRNA pathways not just by methylation. If silencing machineries have broken parts caused by mutations, TEs become active. Thus, there must be an underlying mechanism for maintaining TE integration orientation which is quintessential for conservation of 3D genome architecture.

*(2.2.4) Several members of the host repressive machinery are employed to control TE expression, which includes a variety of small RNA (piRNA pathways), chromatin (KRAB zinc-finger proteins and KAP1/TRIM28-induced H3K9me3 and DNA methylation leading to heterochromatin formation) and DNA modification pathways. In mammals, TEs are primarily silenced through DNA methylation (Fu, Q. & Wang, P. J. Mammalian piRNAs. Spermatogenesis (2014)).*

*piRNA pathways are active in germ cells and silence transposable elements through de novo methylation of transposon promoters (Siomi, M. C., et al. PIWI-interacting small RNAs: the vanguard of genome defence. Nature Reviews Molecular Cell Biology 12, 246–258 (2011)). Defects in members of the mouse PIWI protein family (MILI (also known as PIWI-like protein 2) and MIWI2 (also known as PIWI-like protein 4)) has been shown to activate long interspersed nuclear element (LINE) and long terminal repeat (LTR) retrotransposons in the male germ line, as well as arrest of gametogenesis and complete sterility in males (Carmell, M. A., et al. MIWI2 is essential for spermatogenesis and repression of transposons in the mouse male germline. Dev. Cell 12, 503–514 (2007); Aravin, A. A., et al. Developmentally regulated piRNA clusters implicate MILI in transposon control. Science 316, 744–747 (2007); Kuramochi-Miyagawa, S., et al. DNA methylation of retrotransposon genes is regulated by Piwi family members MILI and MIWI2 in murine fetal testes. Genes Dev. 22, 908–917 (2008)). These studies suggest that piRNAs mainly help in repressing LINE and LTR elements. 70-85% repeat-derived loop anchor CTCF sites in mouse are derived from SINEs, which may be silenced through other host repressive machinery.*

*Moreover, piRNAs repress TE expression and retrotransposition. It is possible that certain TE copies are repressed transcriptionally by these mechanisms but can still function as cis-regulatory CTCF binding sites. The reviewer is absolutely correct in stating that broken parts of the silencing machinery can lead to alleviation of repression and cause TE activation. However, transcriptional activation of TEs is out of the scope of the current study, thus we feel discussion of piRNA is beyond this manuscript.*

*Nevertheless, we would like to once again clarify for both the reviewer and readers that we do not have any reason to believe that there needs to be an underlying mechanism that makes one integration orientation favored over another. Just like mutations, TE insertions happen. And if/when favorable circumstances arise, they may be co-opted by the host.*

*Based on very recent research, one may speculate TE integrations in the vicinity of an existing loop anchor CTCF site could remain in a hypomethylated state and be shielded from host DNA methylation machinery thanks to protection from transcription factors like CTCF (Kremsky, I. & Corces, V. G. Transcription factors protect from DNA re-methylation during reprograming of primordial germ cells and pre-implantation embryos. bioRxiv (2019)). This could be a potential mechanism for maintaining TE integration in its hypomethylated state and if the TE contains CTCF motifs, it could provide redundant binding sites and contribute to the cluster of CTCF sites required for the conservation of 3D genome architecture.*

According to the new analyzed data of orientation of CTCF sites at the left and right loop anchor in mouse CH12-LX and human GM12878 cell line, the author acknowledge the role of orientation of CTCF sites in shaping the 3D genome, especially “when the orientation of the inserted TE is compatible with the local loop structure”, but if TE is not compatible, what will happened?

*(2.2.5) The reviewer puts forward an extremely intriguing and open-ended question. Empirically, our data*

*supports that in the event of a CTCF binding site turnover, if the orientation of the TE-derived CTCF site is compatible with the local loop structure, it is more likely to anchor and perpetuate the loop. However, sometimes TE-derived CTCF sites having incompatible motif orientations can also partake in the turnover process (albeit at a lower frequency) in the absence of other favorably oriented CTCF motifs. This could especially be true for loops that are not exclusively formed by the loop extrusion process that requires*

*convergent CTCF motifs. Indeed, we have already provided this estimate in our original response in (2.1):*

*In mouse, 127 turnover events affecting 123 loops (4 loops had both loop anchors turned-over) were mediated by 124 unique loop anchors (3 turned-over loop anchors tethered 2 loops each). Out of the 124 unique loop anchors, 61 events represent turnover of the left loop anchor and 63 events represent turnover of the right loop. In terms of the orientation of the CTCF motif—for the 61 left loop anchor turnover events, 53 were positive and 8 were negative, and for the 63 right loop anchor turnover events, 45 were negative and 18 were positive (Chi-square test, p-value=5.3x10-11).*

| *mm9 CH12-LX (n=124)* | *Left Loop Anchor* | *Right Loop Anchor* |
| --- | --- | --- |
| *positive CTCF motif* | *53* | *18* |
| *negative CTCF motif* | *8* | *45* |

*In humans, 93 turnover events affecting 89 loops (4 loops had both loop anchors turned-over) were mediated by 84 unique loop anchors (1 turned-over loop anchor tethered 3 loops, and 7 loop anchors tethered 2 loops each). Out of the 84 unique loop anchors, 43 events represent turnover of the left loop anchor and 41 events represent turnover of the right loop. In terms of the orientation of the CTCF motif—for the 43 left loop anchor turnover events, 43 were positive and 0 were negative, and for the 41 right loop anchor turnover events, 40 were negative and 1 was positive (Chi-square test, p-value=3.6x10-19).*

| *hg19 GM12878 (n=84)* | *Left Loop Anchor* | *Right Loop Anchor* |
| --- | --- | --- |
| *positive CTCF motif* | *43* | *1* |
| *negative CTCF motif* | *0* | *40* |

*These results further lend credence to the loop extrusion model and suggest that TE exaptation is more likely when the orientation of the inserted TE (and the underlying CTCF motif provided) is compatible with the local loop structure. We now include these results in the main text.*

*Hence, 26 (8+18) out of 124 (21%) turnover events in mouse and 1 (0+1) out of 84 (1.2%) turnover events in humans are products of CTCF motifs in an orientation that is incompatible with the loop extrusion process.*

*Also, we further discuss it in (2.4):*

*Additionally, we also know that a subset of loops are formed with either divergent CTCF motifs as well as tandem left or tandem right pairs of CTCF motifs (Rao, S. S. et al. A 3D map of the human genome at kilobase resolution reveals principles of chromatin looping. Cell 159, 1665– 1680 (2014); Tang, Z. et al. CTCF-mediated human 3D genome architecture reveals chromatin topology for transcription. Cell 163, 1611–1627 (2015)). Hence, a CTCF binding site turnover event resulting in an opposite orientation motif, may or may not interfere with its underlying function.*

*Nevertheless, a greater understanding of the various types of loops, the diverse factors and mechanistic processes that lead to their formation can help us make better predictions and assessments of the effect of TE-mediated binding site turnover events.*

The author only deleted two inserted TEs which are convergent or tandem to the adjacent CTCF sites, whether TE will maintain the 3D genome architecture while the inserted TEs are divergent to the adjacent CTCF sites? Moreover, deletion of the L1M3f-derived loop anchor merged two neighboring domains, whether the integration site matters at the TAD boundary were not clearly elucidated.

*(2.2.6) We would like to clarify that the two TEs deleted in this study provided CTCF motifs in the convergent*

*orientation. While the L1M3f-derived CTCF site was the loop’s left anchor, the LTR41-derived CTCF site was the loop’s right anchor. We agree with the reviewer that validating more candidate TEs would increase the rigor of the study, however in our opinion these two examples are representative enough to illustrate the potential of TE-mediated CTCF loop anchor/boundary element turnover. In the first example,the ancestral site has decayed, likely allowing L1M3f-derived CTCF site to completely take over the role of providing a loop anchor/TAD boundary. Thus, deletion of L1M3f leads to merging of two neighboring domains. In the second example, the ancestral site is still present albeit is a weaker CTCF binding site relative to the LTR41-derived CTCF site. Thus, deletion of LTR41 leads to a loop anchor/TAD boundary shift to the ancestral site. Our central thesis is that TE-mediated CTCF binding site turnover could help maintain conserved chromatin structure. These examples provide direct support for this central thesis. Once again, we do not intend to use any of our analysis to infer that TEs know where to integrate so that 3D chromatin structure can be maintained.*

If ancestral CTCF binding motif is responsible for maintaining the conserved higher order chromosomal structures, whether inserting a younger TE in vivo will model the evolutionary process?

*(2.2.7) The reviewer puts forward an extremely provocative idea and a direction for potential future experiments. To definitively answer this as well as the previous question (“whether TE will maintain the 3D genome architecture while the inserted TEs are divergent to the adjacent CTCF sites”), we would need to undertake experiments that are beyond the scope of this current manuscript as we would have to replicate conditions that are representative of millions of years of evolution. Moreover, we don’t fully understand all the local chromatin features that differentiate a CTCF motif that is utilized as a loop anchor as opposed to one which is not. In fact, through our study we have only scratched the surface of features (like DNA hypomethylation which we have highlighted in Fig 4A,B) that are required for such events to occur and more fundamental research will certainly help better understand this process.*

*We can only speculate for the reviewer that inserting a younger TE that brings in a compatible CTCF site in the vicinity of an ancestral CTCF site important for maintaining 3D chromatin will merely provide a small likelihood that the TE-derived CTCF site might take over from the ancestral site—thus a small likelihood to influence the evolutionary process. We do not yet have the ability to measure the rate of such event as well as all the variables that impact the process. However, we provide in the current study the discovery of a few hundred such events that span a few dozen million years of evolution in 2 species.*

Minor points:

Too or to “Too add more detail”

*(2.2.8.1)	We apologize for the typographical error. It should read “To add more detail”.*

What’s the “+ve-ve”

*(2.2.8.2)	We apologize for the potential confusion. +ve and -ve are shorthand for positive and negative, respectively. We thank the reviewer for pointing it out and have now replaced it with full text.*

Reviewers’ life could be easier if less undecipherable computer jargons and references are required to “known” before understanding well the flow of logic.

*(2.2.8.3)	We completely agree that jargon should be avoided in all general scientific writing, and have done our best to make the manuscript more legible for a general readership. While we appreciate the reviewers’ concern, we apologize that we have not been able to fix everything that may have hindered the reviewers’ understanding of our paper, since no specific instance was brought up.*

The Supplementary Figure 5 does not contain Sanger sequencing traces.

*(2.2.8.4)	We apologize to the reviewer for the misunderstanding. The Sanger sequencing traces were indeed provided in our previous response. As stated in the original response:*

*(2.3) We thank the reviewer for this request. The precise genotyping was included as a new Supplementary Figure 5, and included below for the reviewer’s reference:*

*We have also submitted the trace (.ab1) files (CRISPR_Genotype_SangerTrace.zip) along with our revision.*

*We included the precise genotype in Supplementary Figure 5 and submitted the Sanger sequencing trace as a supplementary file for the reviewer’s perusal as described above. We now also include screenshots of the region of interest in the trace files for the reviewer’s convenience*

*L1M3f CRISPR clones (DKO1-L2R3, DKO2-L1R1, DKO3-L2R2):*

*LTR41 CRISPR clones (DKO1-L1R1 #1, DKO1-L1R1 #2):*

ChIP-seq data should be provided for the deletion of the LTR41-derived CTCF site, as ancestral MER82 has reduced CTCF binding strength compared to the LTR41 in wild-type cells

*(2.2.8.4)	For the LTR41-DKO GM12878 cell lines, since the sequence corresponding to the LTR41 (which contains the CTCF site) is deleted, we do not expect to see any ChIP-seq signal corresponding to that ~600bp region*

*.*
